# Radioiodine treatment for graves’ disease: a 10-year Australian cohort study

**DOI:** 10.1186/s12902-018-0322-7

**Published:** 2018-12-12

**Authors:** Erin Fanning, Warrick J. Inder, Emily Mackenzie

**Affiliations:** 1 Department of Diabetes and Endocrinology, Princess Alexandra Hospital, Brisbane, Queensland Australia; 2 Faculty of Medicine, the University of Queensland, Brisbane, Queensland Australia; 3 Nuclear Medicine, Department of Radiology, Princess Alexandra Hospital, Brisbane, Queensland Australia

**Keywords:** Graves’ disease, radioiodine., I^131^., Graves’ disease treatment.

## Abstract

**Background:**

Radioactive iodine (I^131^) is a common definitive treatment for Graves’ Disease. Potential complications include worsening, or new development of Graves’ eye disease and development of a radiation thyroiditis. The purpose of the present study was to assess outcomes of patients treated with I^131^ in an Australian tertiary centre over 10 years.

**Methods:**

Data from 101 consecutive patients treated with I^131^ for a diagnosis of Graves’ disease between 2005 to 2015 was collected and reviewed retrospectively. Baseline TSH receptor antibody titre, pre-treatment free thyroxine (FT4), technetium scan uptake, initial treatment, duration of treatment, reason for definitive therapy, complications, and time to remission (defined as euthyroidism or hypothyroidism after 12 months) were recorded.

**Results:**

Of the 92 patients with adequate outcome data, 73 (79.3%) patients achieved remission with a single dose of I^131^. Of the remaining 19 patients, 12 had a second dose and became hypothyroid. TSH receptor antibody titre at diagnosis was significantly lower in the group that achieved remission with the first dose compared with those who did not (*P* = 0.0071). There was no difference in technetium uptake, I^131^ dose, duration of therapy or pre-treatment free thyroxine (FT4). I^131^ was complicated by development of eye disease in 3 patients and 1 (of 11 with pre-existing eye disease) had worsening eye disease. A clinically apparent flare of hyperthyroidism following I^131^ was evident in 8 patients (8.6%).

**Conclusion:**

Radioiodine is an effective therapy for Graves’ Disease with few complications. The majority of patients achieve remission with a single dose. Those who require a second dose are more likely to have higher TSH receptor antibody titres at diagnosis. To the best of our knowledge, this is the first study to report outcomes from radioiodine treatment for Graves’ disease in an Australian population.

## Background

Graves’ disease is the most common cause of adult hyperthyroidism in the developed world.

[1, 2]. It is an autoimmune condition caused by stimulating antibodies acting as an agonist on the thyrotropin (TSH) receptor on thyroid follicular cells [3]. Though it can occur at any age it is most commonly diagnosed in women aged 40–60 years [2]. Treatment options include antithyroid drugs, radioiodine therapy (I^131^) and surgery.

Radioiodine is a safe and effective definitive treatment for Graves’ disease. In Australia, it is generally used as second line therapy for relapsed or persistent disease. I^131^ is taken up by the thyroid gland and incorporated into thyroid hormone, releasing beta particles that cause ionising damage and tissue necrosis. This results in eventual ablation of functional thyroid tissue [1, 2]. On average it takes between 6 to 18 weeks before a euthyroid or hypothyroid state is achieved following I^131^ treatment [2]. Following a single dose of radioiodine, around 15–25% of patients remain hyperthyroid and require additional treatment [1–5]. Hypothyroidism eventually develops in 80–90% of patients. Previous studies have reported factors relating to success of radioiodine including gender (lower remission rates in males) [6], more severe hyperthyroidism [7], thyroid size [8], serum TSH receptor antibody titres [9, 10] and thyroid uptake on radionuclide scans [11].

Potential complications of I^131^ therapy include worsening of Graves’ ophthalmopathy and development of a radiation thyroiditis. I^131^ causes an exacerbation or new occurrence of Graves’ eye disease in 15–20% of patients [1, 11–14]. TSH receptors are found on orbital fibroblasts and are the likely autoimmune target in Graves’ ophthalmopathy. The risk can be mitigated by glucocorticoid prophylaxis in patients with mild disease or patients with multiple risk factors [14, 15]. Early and prompt treatment of hypothyroidism also can prevent the progression of eye disease. Radiation thyroiditis occurs in 1% of patients following radioiodine therapy [2]; the usual onset is within 2 weeks after I^131^ therapy and can be associated with neck tenderness and swelling.

There has been no Australian data regarding the outcome of radioiodine for Graves’ disease previously reported. The aim of this study was to assess the final thyroid status of patients treated with I^131^, including the prevalence and predictors of treatment failure and complications of treatment, in a tertiary endocrinology centre over ten years.

## Methods

Data on consecutive patients treated with I^131^ therapy between 2005 to June 2015 at the Princess Alexandra Hospital (Brisbane, Australia) were retrospectively collected and reviewed. Ethics approval was obtained from the Metro South Human Research Ethics Committee. Consent to participate was not a requirement since this study was considered an audit of practice. Patients were identified from the hospital medical imaging database of all patients who received I^131^ treatment over that time. Those with a confirmed diagnosis of Graves’ disease who received follow up in the hospital outpatient clinic were included. Diagnosis of Graves’ hyperthyroidism was by a suppressed serum TSH (< 0.05 mU/L), along with elevated serum free thyroxine (FT4) and free triiodothyronine (FT3) in association with raised serum TSH receptor antibody titre or a radionuclide scan compatible with Graves’ disease. Patients with other causes of hyperthyroidism (e.g. toxic multinodular goitre, single toxic adenoma) and patients referred from and followed up in other centres were excluded due to lack of data.

Baseline assessment and follow up data were obtained through the computer-based outpatient program ‘Practix’ and supplemented, as required, by patients’ paper charts. Pathology was performed by two private Pathology providers (Sullivan and Nicolaides Pathology, Taringa, Queensland, Australia, or Queensland Medical Laboratories, Murarrie, Queensland, Australia) or the public hospital provider Pathology Queensland, Herston, Queensland, Australia according to patient preference. Parameters including baseline TSH receptor antibody titre, technetium scan uptake, baseline pre-treatment FT4, initial treatment, duration of treatment, complications of medical therapy, reason for definitive therapy, complications of radioiodine treatment, presence of eye disease before and after radioiodine, use of prophylactic glucocorticoids, smoking status and time to hypothyroidism were recorded. The presence and severity of eye disease was assessed individually by the treating physician. The detailed eye examination generally included assessment for conjunctival inflammation, chemosis, periorbital oedema, proptosis, eye movement abnormalities or diplopia and any evidence of visual loss. Clinical severity was graded as mild, moderate or severe. Treatment failure was defined as persistent hyperthyroidism at 12 months post I^131^ requiring either long-term thionamide therapy, a repeat dose of I^131^ or thyroidectomy.

I^131^ (sodium iodide powder in prefilled capsules) was administered orally in the Department of Nuclear Medicine at the Princess Alexandra Hospital. A fixed dose (administered activity) of 500 MBq is currently used. In earlier years of the study, this had been 450 MBq and some patients received a lower dose due to the practice of an individual nuclear medicine physician. Antithyroid drugs were ceased 3–5 days prior to and recommenced 5 days following I^131^ treatment as needed. All female patients of childbearing age underwent a pregnancy test (serum beta hCG) prior to proceeding with therapy. Patients were subsequently followed up with thyroid function tests 4–6 weekly after treatment [2]. Remission was defined as hypothyroidism or euthyroidism within 12 months of a single dose RAI.

Continuous data failed parametric assumptions and therefore are presented as median and 95% confidence intervals (CI). Categorical variables are presented as simple proportions (%). A Mann Whitney U test was used to compare continuous baseline variables between groups. A *P* value of < 0.05 was considered significant. All data analysis was performed using Graphpad Prism 7.03.

## Results

One hundred and one eligible patients were identified, 74 (73%) of whom were female. The initial medical therapy was carbimazole in 93 (92%) patients, 6 used PTU (6%) and 2 (2%) patients did not receive medical therapy prior to undergoing I^131^. During the course of treatment, 17 patients changed therapy from carbimazole to PTU and 1 patient was changed from PTU to carbimazole. An adverse reaction to the antithyroid drug was reported in 29 (28.7%) patients; 19 secondary to carbimazole and 10 due to PTU. The primary indication for definitive treatment was disease relapse following a trial withdrawal of antithyroid drug therapy in 41 patients, a poor response to medical therapy in 29 patients due and intolerance/complications of treatment in 18 patients (Table 1). The most common adverse reaction to medical therapy was a rash in 8 patients, followed by neutropaenia (neutrophils < 1.0 × 10^9^/L) in 6 patients. LFT derangement was reported in 3 patients taking PTU and 1 patient taking carbimazole.

**Table 1:** Reasons for definitive therapy

Reason for definitive therapy	Number
Relapse	41
Poor response/unable to wean medication	29
Intolerant/complications of medical therapy	18
Patient compliance	4
Carbimazole unavailable	2
Remote location	2
Patient preference	2
Likely to require multiple contrast loads	1
Hepatitis C on interferon	2
Cardiovascular comorbidities	4

Baseline characteristics are presented in Table 2. Pre-existing eye disease was present in 11 patients; four of these were documented as mild/inactive. The median duration of medical therapy was 24 months prior to receiving radioiodine therapy. The range was 3 weeks to 12 years. A few outliers received a very long duration of medical therapy. The reasons for this included previous lack of ongoing specialist input or prior hesitance on behalf of the patient to undergo definitive therapy. The median TSH receptor antibody titre at diagnosis was 8 IU/L (range 0–240). Fifteen (15%) patients were documented to be smokers. Of the 11 patients who had documented eye disease prior to radioiodine therapy, 7 received prophylactic corticosteroids. The administered activity of I^131^ was between 311 and 580 MBq (mean 495.7 MBq).

**Table 2:** Baseline characteristics

Baseline Characteristics	
Pre-existing eye disease	11 (4 documented as mild/inactive)
Duration of Medical Treatment (months)	24 (3 weeks to 12 years)
Technetium uptake (%)	4.3 (0.8–66)
TSH receptor antibody (IU/L)	8 (0–240)
Smoker (documented)	15
Glucocorticoid cover with pre-existing eye disease	7

Data are expressed as median (range)

**Fig. 1:**
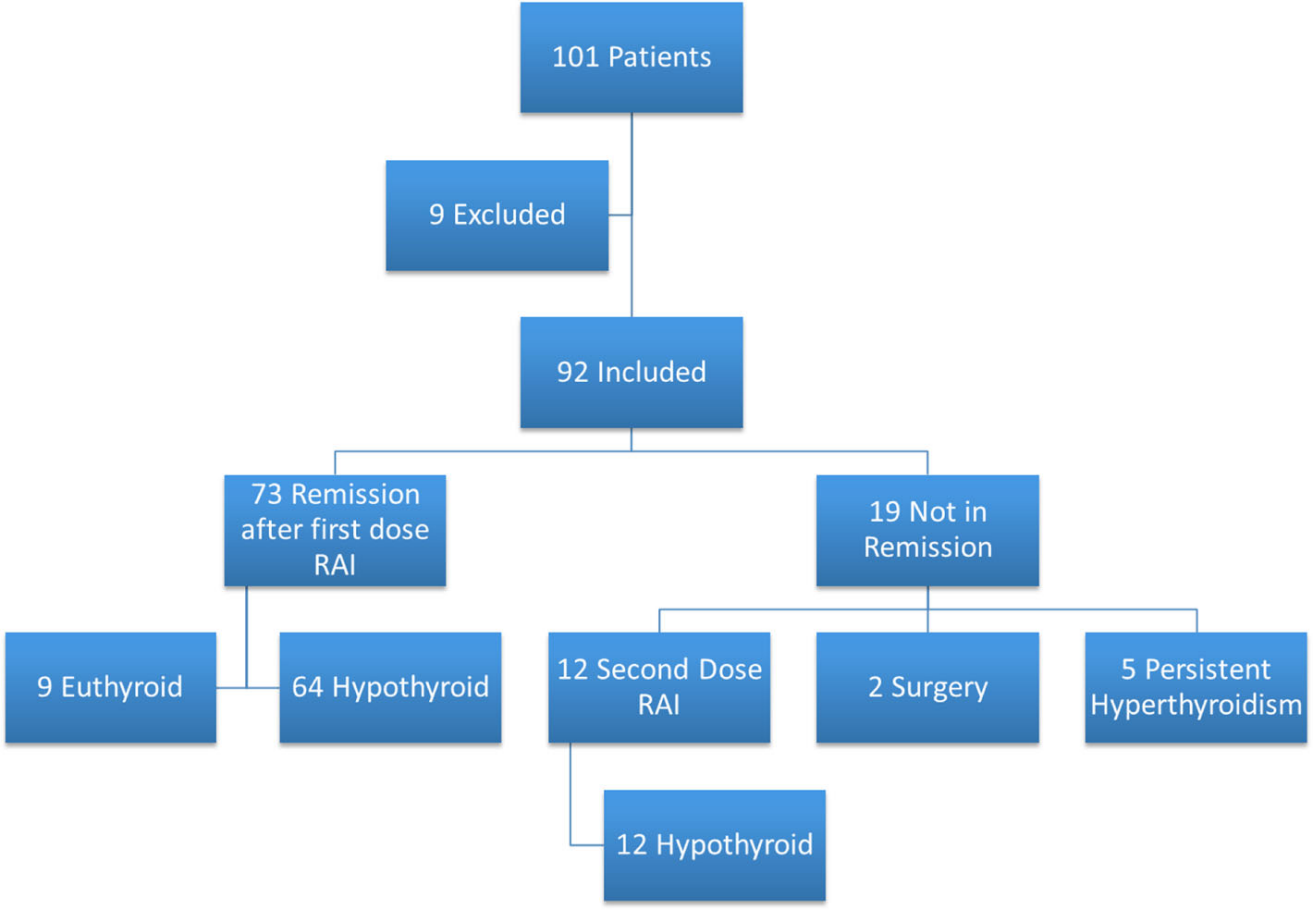
Outcomes in 101 patients with Graves’ disease treated with radioactive iodine

Regarding outcomes of radioiodine therapy, 92 patients had adequate follow up data for inclusion (Fig. 1). Remission following a single dose of I^131^ was achieved in 73 (79.3%) patients. Of the 19 patients who did not achieve remission, 12 had a second dose and became hypothyroid, 2 underwent surgery and 5 had persisting hyperthyroidism requiring medical therapy. Of the patients who achieved remission with a single dose of I^131^, 64 patients became hypothyroid (87.6%) and 9 patients (12.3%) remained euthyroid. The median time from I^131^ administration to hypothyroidism was 4 months [3, 4].

TSH receptor antibody titre at diagnosis was significantly lower in the group that achieved remission with the first dose compared to those who did not (*P* = 0.0205). There was no difference in technetium uptake, I^131^ administered activity, duration of medical therapy, pre-treatment FT4 or duration of disease (Table 3).

**Table 3:** Factors potentially implicated in response to radioiodine treatment

	Remission (*n* = 73)	No Remission (*n* = 19)	P
TSH receptor antibody (IU/L)^a^	7.2 (4.5–9)	18 (5.4–68)	0.02
Technetium Uptake (%)	4.3 (3.1–5.2)	3.5 (1.8–7.1)	0.35
I^131^ administered activity (MBq)	500 (500–511)	501 (499–505)	0.58
Duration of medical therapy (months)	21 (12–24)	36 (6–48)	0.31
Duration of disease (years)	3 (2–4)	3 (1–4)	0.88
Pre-Treatment FT4 (pmol/L)	34 (27–39.9)	28.9 (19.6–42)	0.33

Data expressed as median (95% CI)

Remission is defined as hypothyroidism or euthyroidism within 12 months of a single dose RAI

^a^TSH receptor antibody titre at diagnosis

Radioiodine therapy was complicated by new onset of eye disease in 3 (3.3%) patients (Table 4). In each of these patients, the eye disease was documented as mild and did not require any treatment. Of the 11 patients with pre-existing eye disease, one developed worsening of their eye disease which was severe and eventually required surgical decompression, long term glucocorticoid therapy and radiotherapy. This patient was documented as having inactive eye disease prior to therapy and therefore did not receive prophylactic glucocorticoids. A clinically significant flare of hyperthyroidism following radioiodine was evident in 8 patients (8.6%).

**Table 4:** Complications of radioiodine

Complication	Number (%)
Worsening of pre-existing eye disease	1 (1.1%)
Severe eye disease requiring decompression	1 (1.1%)
Worsening of eye disease despite prophylactic glucocorticoids	0
Flare of clinically significant hyperthyroidism	8 (8.6%)
New onset eye disease	3 (3.3%)
Failure to achieve remission with single dose of RAI	19 (20.7%)

## Discussion

This study assessed the outcomes of patients treated with I^131^ for Graves’ disease at an Australian tertiary hospital over 10 years. We found that 79% of patients achieved remission with a single dose of I^131^. Of the patients who did not achieve remission with the first dose of radioiodine, all those treated with a second dose became hypothyroid. Individuals who did not achieve remission with a single dose were more likely to have higher TSH receptor antibody titres at diagnosis. There was a low rate of complications associated with radioiodine. Only 4.3% of patients developed new-onset or worsening of eye disease and 8.6% developed a transient flare of clinically significant hyperthyroidism. To our knowledge, this is the first study that has reported outcomes of I^131^ therapy for Graves’ disease from an Australian centre.

The remission rate following I^131^ in our study is similar to previously published studies. Metso et al. reported a remission rate of 74% with a single dose of I^131^ for treatment of Graves’ disease in a prospective cohort study in Finland [4]. Zantut-Wittmann et al. reported a 37.8% rate of persistent hyperthyroidism 12 months following radioiodine therapy [5]. A fixed dose of 370 MBq was used in this study, in comparison to our centre where the mean administered activity was higher at 495.7 MBq. Some studies have reported higher remission rates of up to 93% [7, 16], however these included patients treated with I^131^ as first-line therapy. In our centre, patients usually undergo an initial trial of antithyroid medication; most are referred for I^131^ in the event of relapse or treatment failure and thus are likely to have more resistant disease.

Consistent with other studies, we found a significant difference in TSH receptor antibody titre at diagnosis comparing patients who achieved remission with a single dose of I^131^ to those who remained hyperthyroid. Murakami et al. found that TSH receptor antibody activity immediately before radioiodine therapy was significantly higher in patients who did not achieve remission with a single dose of I^131^ compared to those who did [9]. Chiovato et al. also found that pre-treatment TSH receptor antibody titres were significantly higher in patients who remained hyperthyroid post I^131^ than in those that became hypothyroid or euthyroid [10]. Our study assessed the TSH receptor antibody titre at diagnosis, rather than pre-treatment as it was consistently available. Based on the above findings, the measurement of TSH receptor antibody titres at the onset of disease and prior to definitive treatment may be a useful tool to help predict patients who may be less likely to achieve remission with a single dose of radioiodine. This can assist with counselling patients prior to treatment. Nearly all patients in our study received antithyroid drugs prior to treatment as is standard management for Graves’ disease in Australia. Prolonged treatment with antithyroid drugs reduces the serum TSH receptor antibody level [17]. Given that almost all patients were pre-treated with antithyroid drugs in our study it was not possible to look at the relative impact of the TSH receptor antibody titre at diagnosis and after a long period of antithyroid drug treatment on the success of radioiodine therapy, however this may be a direction for future research.

There was no difference in Technetium uptake, duration of disease, duration of medical therapy, severity of disease (measured by pre-treatment FT4) or administered activity of I^131^ between patients who achieved remission and those who did not. In contrast, Zantut-Whitmann et al. found that patients with a [^99m^Tc] pertechnetate uptake of ≥12.5% had a 4.1 times increased risk of persistent hyperthyroidism [4]. The authors of this study also reported that thyroid mass < 53.7 g had an 8.9 fold higher probability of treatment success [11]. The relationship between thyroid volume and treatment success has also been reported in other studies, with a larger thyroid volume prior to I^131^ being associated with a reduced chance of treatment success [10, 18]. Data regarding thyroid volume was not included in this study as it was not consistently available.

Radioiodine is associated with the development or worsening of thyroid eye disease in about 15–20% of patients [3, 13, 14, 19]. Traisk et al. assessed the incidence of Graves’ ophthalmopathy in patients randomised to either 18 months of medical treatment, or I^131^ therapy. Worsening or development of new-onset eye disease was significantly more common in the I^131^ group (38.7%) compared with the medical treatment group (21.3%) [13]. Bartalena et al. found that radioiodine treatment was often followed by an exacerbation of eye disease in at least half of patients with pre-existing ophthalmopathy [15].

In our cohort, only four patients developed new-onset or worsening of pre-existing eye disease (4.3%), of whom three developed new-onset eye disease. The eye disease was documented to be mild in each case and did not require any treatment. Of these patients, one was a smoker and another was documented to be an ex-smoker. The occurrence of new or worsening eye disease was lower in our cohort than previously reported. This may be explained by several factors including: i) a low rate of pre-existing eye disease (11 patients; 11%); ii) careful patient selection excluding patients with more significant eye disease for I^131^; and, iii) the use of prophylactic glucocorticoids. The one patient who subsequently developed severe eye disease was a smoker and was documented to have mild, inactive disease prior to therapy and thus did not receive prophylactic glucocorticoids. The TSH receptor antibody titre of this patient was below the mean at 14 U/L.

Other risk factors for the development or progression of ophthalmopathy following radioiodine such as smoking, high levels of pre-treatment serum T3 and post radioiodine hypothyroidism were not examined in the present study due to the low number of patients affected.

Prophylactic glucocorticoids have been shown to be highly effective in reducing the risk of thyroid eye disease in patients treated with I^131^ [15, 16, 19]. In a systematic review, no patients with mild eye disease treated with prophylactic glucocorticoids prior to I^131^ developed worsening of their pre-existing eye disease [20]. Consistent with this, none of the seven patients in our study treated with prophylactic glucocorticoids for pre-existing mild eye disease developed any exacerbation.

It is important to recognise several limitations of this study. Firstly, this is a retrospective study based on chart review. Data for variables such as TSH receptor antibody status were not taken at a consistent time point for each patient and some data (e.g., smoking status), were inconsistently reported. The presence or absence of ophthalmopathy was dependent upon accurate assessment and documentation by the treating clinician and therefore, transient or mild ophthalmopathy may have been missed. This may partly explain the lower rates of new onset or worsening eye disease in our group compared to other studies. The assessment and clinical grading of eye disease is likely to have been variable between clinicians. TSH receptor antibody assays have changed over the included study period and were measured by three different laboratories.

## Conclusion

In conclusion, the first published Australian series has confirmed radioiodine is a safe and effective definitive treatment for Graves’ Disease. Most patients become hypothyroid following a single dose of I^131^, with a single dose of radioiodine resulting in long-term remission from Graves’ disease in 79%. Of the patients who remained hyperthyroid after the first dose of radioiodine, all those treated with a second dose achieved remission. With careful patient selection, there was a low rate of complications associated with I^131^ therapy.

## Abbreviations

FT4: Free thyroxine
TSH: Thyroid stimulating hormone
